# Biological Networks Underlying Abiotic Stress Tolerance in Temperate Crops—A Proteomic Perspective

**DOI:** 10.3390/ijms160920913

**Published:** 2015-09-01

**Authors:** Klára Kosová, Pavel Vítámvás, Milan Oldřich Urban, Miroslav Klíma, Amitava Roy, Ilja Tom Prášil

**Affiliations:** 1 Laboratory of Plant Stress Biology and Biotechnology, Division of Crop Genetics and Breeding, Crop Research Institute, Drnovská 507/73, 16106 Prague, Czech Republic; E-Mails: vitamvas@vurv.cz (P.V.); olinek.vcelar@seznam.cz (M.O.U.); klima@vurv.cz (M.K.); prasil@vurv.cz (I.T.P.); 2 Research Institute of Agricultural Engineering, Drnovská 507, 16106 Prague, Czech Republic; E-Mail: roy.amitava@vuzt.cz

**Keywords:** abiotic stresses, temperate crops, proteomics, protein functions, stress tolerance, multiple stress, protein markers

## Abstract

Abiotic stress factors, especially low temperatures, drought, and salinity, represent the major constraints limiting agricultural production in temperate climate. Under the conditions of global climate change, the risk of damaging effects of abiotic stresses on crop production increases. Plant stress response represents an active process aimed at an establishment of novel homeostasis under altered environmental conditions. Proteins play a crucial role in plant stress response since they are directly involved in shaping the final phenotype. In the review, results of proteomic studies focused on stress response of major crops grown in temperate climate including cereals: common wheat (*Triticum aestivum*), durum wheat (*Triticum durum*), barley (*Hordeum vulgare*), maize (*Zea mays*); leguminous plants: alfalfa (*Medicago sativa*), soybean (*Glycine max*), common bean (*Phaseolus vulgaris*), pea (*Pisum sativum*); oilseed rape (*Brassica napus*); potato (*Solanum tuberosum*); tobacco (*Nicotiana tabaccum*); tomato (*Lycopersicon esculentum*); and others, to a wide range of abiotic stresses (cold, drought, salinity, heat, imbalances in mineral nutrition and heavy metals) are summarized. The dynamics of changes in various protein functional groups including signaling and regulatory proteins, transcription factors, proteins involved in protein metabolism, amino acid metabolism, metabolism of several stress-related compounds, proteins with chaperone and protective functions as well as structural proteins (cell wall components, cytoskeleton) are briefly overviewed. Attention is paid to the differences found between differentially tolerant genotypes. In addition, proteomic studies aimed at proteomic investigation of multiple stress factors are discussed. In conclusion, contribution of proteomic studies to understanding the complexity of crop response to abiotic stresses as well as possibilities to identify and utilize protein markers in crop breeding processes are discussed.

## 1. Introduction

Abiotic stress factors, especially cold (low temperatures), drought and salinity, profoundly affect crop growth and development in temperate climate zones which can be defined as regions lying between tropical and polar zones, *i.e.*, between the Tropic of Cancer and the Arctic Circle in the northern hemisphere and between the Tropic of Capricorn and the Antarctic Circle in the southern hemisphere, respectively, with relatively moderate temperatures and significant temperature differences between summer and winter seasons [1]. The major crops grown in temperate climate include cereals such as common wheat (*Triticum aestivum*), durum wheat (*Triticum durum*), barley (*Hordeum vulgare*) and maize (*Zea mays*), potato, leguminous plants such as soybean and alfalfa, sugar beet, oilseed rape, flax, sunflower, tobacco, vegetables such as chicory, pea, tomato and watermelon, and woody crops such as grapevine. Abiotic stress factors represent the major constraints limiting agricultural production and reducing crop yield. Under global climate change, the risk of damaging effects of abiotic stress factors on crop production increases. The risk includes not only an increased aridization and salinization in several regions such as Australia, the Middle East, and southern Europe (Spain), but also an increased frequency of frost damage in temperate areas due to rapid temperature shifts during winter and early spring seasons as a consequence of freeze-thaw cycles [2].

**Figure 1:**
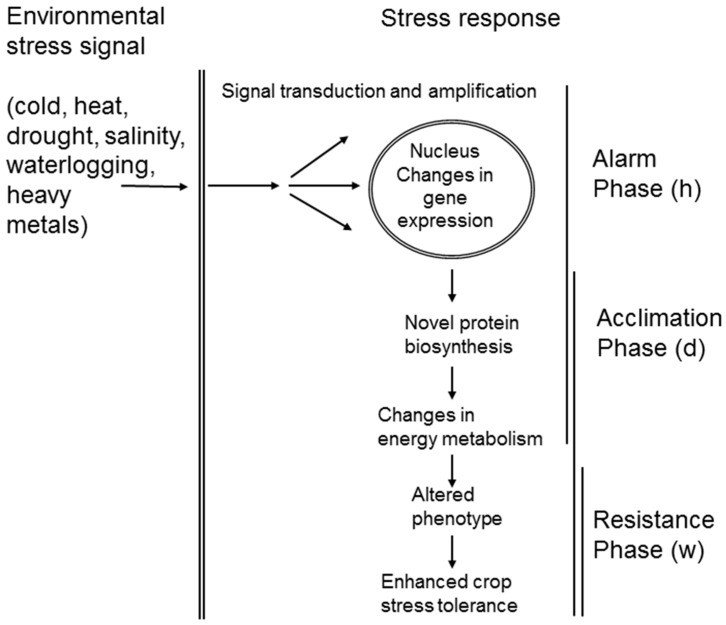
A schematic representation of the dynamics of plant stress perception and stress response at cellular level. The first phase of plant stress response, an alarm phase, is usually very short (hours; h) with respect to the following acclimation phase (days; d) and resistance phase (weeks; w). There are also significant overlaps between the individual processes and phases with respect to their timing.

As living organisms, plants tend to maintain homeostasis in their bodies. Plants are poikilothermic organisms, *i.e.*, they cannot actively regulate temperature of their bodies. Basic principles of plant water uptake from soil are also passive as they are governed by differences between soil and plant cell water potential. However, plants actively exchange energy, water, and thousands of chemical compounds between themselves and the environment. Therefore, plants sense changes in their environment and respond to them in order to prevent damage of their bodies (Figure 1). Plant stress response is a dynamic process in which several phases could be distinguished—an alarm phase, an acclimation phase, a resistance phase, an exhaustion phase when stress lasts too long or is too severe, and a recovery phase after a cessation of a stress factor which leads to an establishment of a novel homeostasis [3,4,5]. Each phase of plant stress responses is aimed at an establishment of novel homeostasis under altered environmental conditions and is therefore accompanied by profound alterations in plant cellular composition. Recently, a boom of high-throughput separation and identification techniques has enabled researchers to study plant cellular responses in a more complex way using so-called “omics” approaches including structural and functional genomics, transcriptomics, proteomics, and metabolomics. Proteins represent a crucial component of plant stress response since they are directly involved in plant cell structure and metabolism [5]. They are products of genes, but they are much closer to the resulting phenotype since they act as direct effectors of the phenotype, *i.e.*, they constitute plant cell structure and actively participate on metabolism of all cellular components. The total of all proteins in a given tissue at a given time—proteome—is uniquely variable. Unlike the genome, which is only one for a given organism, there are infinite proteomes which depend on an organism’s growth and developmental stage, plant tissue, and cell type as well as on ambient growth conditions. Moreover, one gene can give rise to various protein products due to mechanisms of posttranscriptional (alternative RNA splicing, RNA editing, *etc.*) and posttranslational modifications (PTMs—phosphorylation, acetylation, methylation, ubiquitination, myristoylation, *etc.*). Therefore, the total number of distinct proteins synthesized by a given organism can be several orders higher than the total number of genes encoded by a genome of the given organism. Considering the splendid variety of proteomes, a plant thus possesses an efficient tool to modulate its response to specific environmental conditions.

During the past two decades, high-throughput plant proteomic studies have revealed a boom due to technological advancement in both gel-based and gel-free protein separation and relative quantification techniques (2D-DIGE, iTRAQ, label-free MS/MS protein quantification) and publication of complete genome sequences of model plants (*Arabidopsis thaliana*) as well as major crops (rice, potato, soybean, maize, barley, common wheat). These factors enabled the researchers to study plant responses to internal and external (environmental) factors including abiotic stresses at proteome level. Study of proteome response of major crop plants to environmental stress is of high interest due to elucidation of biological mechanisms and identification of crucial proteome components underlying an enhanced crop stress tolerance. Results of proteomic studies dealing with proteome response to major abiotic stress factors in temperate crops were already reviewed in several papers including large-scale comprehensive reviews on proteomics of abiotic stresses in crop plants [5,6] as well as more specialized reviews dedicated to specific stress factors, crops or cellular fractions such as reviews on salinity proteomics [7,8,9], subcellular proteomics of crop plants exposed to stress [10], proteomics of heavy metal stress [11], proteomics of abiotic stresses in wheat and barley [12,13], soybean proteomics [14], proteomics of flooding stress in soybean [15], and others. The aim of the present review paper is to summarize recent results of proteomic studies obtained in major crop plants grown in temperate climate regions and subjected to major abiotic stresses—drought, salinity, cold, frost, waterlogging, and heavy metal stress. The major focus of the review is on contribution of proteomic studies to elucidation of biological mechanisms underlying stress response in temperate crops, with special attention paid to proteins revealing differential responses between crop genotypes with differential stress tolerance levels as well as proteomic studies dealing with the effects of multiple stress factors. In conclusion, future challenges in proteomic studies focused on elucidation of protein roles under stress are discussed and possible applications of proteomic results in crop breeding programs aimed at an improvement of crop stress tolerance are suggested.

## 2. A Brief Summary of Proteomic Studies on Stress Response in Temperate Crops

Common wheat (*Triticum aestivum*) is the most grown crop worldwide with the third highest total production of *ca.* 716 million metric tons [16] and its production is still dominant in temperate climate zone although an introduction of photoperiod-insensitive genotypes has enabled wheat production also in tropical zones. Maize, potato, and barley are the second, fourth, and fifth most produced crops worldwide, respectively, with a dominant production also in temperate climate zone.

Unlike model plants such as *Arabidopsis thaliana* and rice, a complete genome annotation is not available for many crops although recently, draft genome annotations were published for major crops grown in temperate climates including maize [17], potato [18], barley [19], common wheat [20], and soybean [21], common bean [22], tobacco [23], tomato [24], oilseed rape [25], sugar beet [26], watermelon [27], and grapevine [28].

Most proteomic studies have dealt with most cultivated cereal crops including common wheat, barley, maize, potato, and soybean whose whole genome sequences are already publicly available. However, several proteomic studies on other temperate crops are being published including field crops such as durum wheat [29,30], Indian mustard (*Brassica juncea*) [31], sunflower [32,33], flax [34,35], leguminous crops such as alfalfa [36,37,38], and white lupin [39], and vegetables such as chicory [40], and pea [41,42]. The major abiotic stresses studied include temperature stress (cold, frost, heat), water stress (drought, waterlogging), osmotic stress (polyethylene glycol—PEG), salinity, imbalances in mineral nutrition, and heavy metal stress. An overview of proteomic studies on temperate crops subjected to abiotic stresses listed above is given in Table S1.

## 3. Dynamics of Crop Stress Response at Proteome Level

### 3.1. Alarm Phase

#### Stress Signaling and Gene Expression

An ambient cue is recognized by a plant cell as a signal when it leads to significant changes in physical properties (changes in the fluidity of phospholipid molecules in plasma membrane bilayer under low or high temperatures) or chemical composition of the ambient environment (e.g., dehydration stress or salinity leading to a decrease of water potential or an increase in Na^+^ concentration in ambient soil solution). The changes in plasma membrane physico-chemical properties lead to conformational alterations of plasma membrane-associated peripheral or integral signaling protein complexes (e.g., two-component histidine kinases with respect to cold [43,44]; SOS1/SOS2/SOS3 complex in Na^+^ signaling; reviewed in [45,46]). The initial signal is then transferred and amplified by several second messengers to the nucleus where the signal induces changes in gene expression leading to alterations in plant transcriptome, proteome and metabolome underlying an active plant stress response. At proteome level, especially when using 2DE based approaches, alterations in protein abundance are scarcely detected due to a relatively low abundance of signaling proteins with respect to other cellular proteins and due to the rapidity of their changes during an alarm phase of stress response. However, at least some signaling proteins such as components of mitogen-activated protein kinase (MAPK) cascade, calcium signaling (calmodulin, calnexin), components of heterotrimeric plasma membrane-located G proteins, phospholipases C and D (PLC, PLD), were detected in stress-treated plants, especially under drought and salinity [47,48,49]. It was proposed that Ca^2+^ signaling may affect cellular Na^+^/K^+^ homeostasis via SOS1/SOS2/SOS3 complex [45] and plays also an important role in sensing of osmotic stress [50]. Phospholipases cleave small molecules from plasma membrane phospholipid heads which then act as second messengers. Protein phosphorylation plays an important role in the activity of several signaling proteins—a differential phosphorylation level of not only signaling proteins (MAPK, calcium-dependent protein kinase CDPK, sucrose non-fermenting-related kinase SnRK2, protein phosphatase PP2C), but also transcription factors (ABI5), transport proteins (aquaporins, H^+^-ATPase) and protective proteins (COR/LEA) was found in drought-treated wheat [51] and PEG treated common bean [52]. The 14-3-3 proteins are known as regulatory proteins which can bind several signaling proteins, cell cycle regulating kinases and ion transporters (H^+^-ATPase, K^+^ channels) depending on their phosphorylation status; 14-3-3 proteins thus significantly modulate plant stress response [53]. An increase in 14-3-3 proteins was found in copper- and water-stressed wheat [37,54,55,56,57,58], barley [59], in PEG-stressed soybean plasma membrane fraction [50], in salt-stressed maize [49], and others.

In the nucleus, the signal is transformed into the changes in gene expression. Changes in several transcription factors as well as other regulatory proteins such as glycine-rich RNA binding proteins or lectins such as VER2 were found in proteomic studies aimed at wheat response to a long-term cold treatment affecting plant development [60,61,62,63]. An increase in bHLH transcription factor was found in salt-stressed soybean seedlings [64]. The abundance and activity of several transcription factors and regulatory proteins is modulated by several phytohormones upregulated during the alarm phase of stress such as ABA (AREB/ABF; MYB, MYC transcription factors), JA (glycine-rich RNA binding proteins, lectin VER2), SA, and others [62,65].

### 3.2. Acclimation Phase

#### 3.2.1. Protein Metabolism

Changes in gene expression are coupled with changes in protein metabolism including both protein biosynthesis and degradation. Plant adjustment to an altered environment requires myriads of novel proteins to be synthesized as well as myriads of proteins to be degraded. Therefore, several changes in the abundance of ribosomal proteins include proteins belonging to both eukaryotic and prokaryotic-type (mitochondrial and plastidic) ribosomal subunits. For example, an increase in ribosomal protein L39 involved in accuracy of translation in drought-treated grapevine was found by Vincent *et al.* [66]. A decrease in chloroplast 30S ribosomal protein S10 indicates a down-regulation of chloroplast protein biosynthesis in salt-sensitive canola cultivar Sarigol since protein S10 seems to be crucial for tRNA binding to ribosomal surface and the stability of 30S ribosomal subunit [67]. Moreover, differential abundance of several eukaryotic translation initiation and elongation factors was found. However, the observed change may be related also to processes other than protein biosynthesis. For example, eukaryotic translation initiation factor 5A (eIF5A) reveals multiple regulatory roles in cell cycle regulation—different forms of eIF5A are proposed to affect a switch between cell proliferation and cell death [68]. A lower decrease of eIF5A3 isoform in salt-treated *Triticum aestivum* × *Thinopyrum ponticum* hybrid with respect to its *T. aestivum* parent indicates a higher anti-senescence ability of salt-tolerant hybrid compared to salt-sensitive parent under salinity [54]. Factor eIF5A was also detected in salt-tolerant oilseed rape Hyola 308 while it was absent in salt-sensitive cultivar Sarigol under salt stress [67]. Moreover, alterations in several regulatory proteins involved in mRNA stabilization, processing, and editing, e.g., nuclear-encoded chloroplastic ribonucleoprotein cp29, were found in stressed plants [69,70]. Regulation of cell cycle and programmed cell death (PCD) is also associated with translationally controlled tumor protein homolog (TCTP) which has been characterized as an agent decreasing cytosolic Ca^2+^ levels and thus inhibiting PCD [71]. An increased abundance of TCTP was observed under several stresses including salinity [72], drought [56], and others.

Protein degradation pathways include mainly proteins associated with protein targeting by ubiquitin to proteasome degradation. An increase in proteasome subunits, e.g., 20S proteasome alpha [56,61,73] and alterations in E2 ubiquitin ligase indicating an up-regulation of proteasome-dependent protein degradation were found in cold-treated winter wheat [62].

Alterations in protein metabolism also affect alterations in amino acid metabolism. Several amino acids represent not only protein components, but also precursors of various stress-related compounds and key components of metabolic pathways associated with carbon and nitrogen metabolism. For example, glutamate (glutamic acid) and glutamine represent crucial compounds associated with nitrogen assimilation, glutathione and proline biosynthesis, phenylalanine and tyrosine can be deaminated to yield *trans*-cinnamic acid and *p*-coumaric acid, precursors of lignin components synthesized via the phenylpropanoid pathway, methionine is a precursor of *S*-adenosylmethionine (SAM) which is not only a universal methyl donor in plant cells, but also a precursor of many stress-related compounds including phytosiderophores (synthesized from SAM in a series of reactions known as Yang cycle), polyamines (spermine, spermidine, putrescine), ethylene and other stress-related compounds. An increase in glutamine synthetase in drought-treated soybean roots and alfalfa leaves, respectively, was consistent with an enhanced content of proline and a decreased osmotic potential [36,74]. An increase in methionine synthase or SAM synthase (SAMS) was found in many proteomic studies dealing with various stress factors including cold [62,75], drought, salinity [76,77,78], *etc.* Alterations in enzymes involved in phytosiderophore biosynthesis were also found in some studies, e.g., an increase in methylthioribose kinase was found in boron-treated barley [79], while a decrease in IDI2, IDS2, and IDS3 proteins involved in a biosynthesis of mugineic acid (a precursor of phytosiderophores) was found in salt-treated barley [80] indicating a reduction of metal uptake as potential catalyzers of ROS. In contrast, an increase in ferritin levels was found in flax cell culture exposed to elevated cadmium [34] and wheat leaves exposed to salinity [81]. Moreover, several amino acids can be deaminated by aminotransferases to yield oxoacids, which are intermediates of Krebs cycle, a crucial pathway of aerobic respiration.

#### 3.2.2. Energy Metabolism

An active plant response to stress is associated with enhanced demands on energy. Therefore, alterations in energy metabolism were reported in several proteins involved in energy metabolism. Adenosine trisphosphate (ATP) represents an immediately available energy source which functions as a cofactor in several energy-demanding reactions. Alterations in enzymes involved in a cleavage of macroergic phosphate bonds such as nucleoside diphosphate kinase (NDPK) were reported in several papers [40,69,75,82,83]. Novel ATP molecules are synthesized in photosynthesis, anaerobic, and aerobic respiration. Thus, alterations in both mitochondrial and chloroplast ATP synthases subunits, especially α, β, γ and ε subunits of chloroplast and mitochondrial CF1 complex directly involved in ATP biosynthesis, are reported in relation to stress [36,48,70,84,85,86,87]. An active site of ATP synthesis lies in β subunit of CF1 ATP synthase complex which was reported to be declined under drought in wheat [88].

Photosynthesis is highly sensitive to imbalances between primary electron-transport processes and secondary chemical reactions associated with CO_2_ assimilation. A discrepancy between the rate of primary and secondary photosynthetic reactions enhances a risk of ROS formation. Changes in photosystem-associated proteins, especially OEE proteins as components of PSII OEC center involved in photolysis of water, were found in several studies [29,56,60,77,83,86,89]. A decrease in RubisCO large and small subunits (RubisCO LSU and SSU) as well as Calvin cycle enzymes phosphoglycerokinase (PGK), phosphoribulokinase (PRK), and transketolase was found in drought- and salt-treated durum wheat [29,30] as well as in cold-treated spring Iranian wheat Kohdasht [60]. In contrast, an increase in proteins with protective functions such as RubisCO activase A [29,30,76,90], a Triticeae-specific thermostable RubisCO activase B [91], and carbonic anhydrase [30,56], were found in stressed plants. Changes in OEE1 and OEE2 proteins were frequently found in salt-treated barley [76,77], durum wheat [29], and tobacco [92], and an increase in OEE1 protein was observed in drought-treated barley infected by *Piriformospora indica* [56]. Proteomic studies have usually shown an increase in OEE proteins under milder stress and in tolerant plant materials while a decrease under severe stress or in sensitive plant materials [93,94]. Moreover, an increase in RubisCO chaperones CPN60-α, CPN60-β, and 20-kDa co-chaperonin was found under several stresses [29,55,62,69,95,96,97]. Stress also leads to profound changes in aerobic metabolism. Due to an enhanced risk of ROS formation, a decrease in some components of mitochondrial electron-transport chain such as NADH-dependent ubiquinone oxidoreductase was found in some studies [83,95]. Imbalances in the rates of several aerobic processes lead to an enhanced risk of oxidative stress. Therefore, an increase in alternative electron-transport pathways using alternative oxidase [98,99] as well as in enzymes involved in anaerobic ATP-producing processes such as glycolysis (glyceraldehyde-3-phosphate dehydrogenase GAPDH, triosephosphate isomerase TPI, enolase ENO, pyruvate kinase) and alcoholic fermentation (alcohol dehydrogenase ADH, aldehyde dehydrogenase, formate dehydrogenase) has been found in several studies dealing with waterlogged root cells, but also with seedlings and young plants exposed to severe dehydration stresses such as drought and salinity [29,30,62,73,83,95,100]. In contrast, a decline in the levels of glycolytic enzymes was found during the flax seed development in radioactivity-contaminated environment [35]. However, an increased need for energy can lead to an increase in several proteins related to aerobic metabolism (Krebs cycle, mitochondrial electron-transport chain) as indicated by an enhanced level of thiamine thiazole synthase, dihydrolipoamine acetyltransferase, and other cofactors of dehydrogenase complex catalyzing pyruvate conversion to acetyl-CoA [101]. Differential patterns of changes in several isoforms of glycolysis enzymes were frequently reported in one proteomic experiment as shown for enolase isoforms in drought-treated sunflower roots [33]. Moreover, nuclear isoforms of cytoplasmic glycolytic enzymes can act as important regulators of stress-responsive pathways as reported for a nuclear isoform of ENO encoded by *LOS2* locus and involved in regulation of cold-inducible CBF pathway [102] or for a nuclear GAPDH involved in tRNA transport [103,104].

An enhanced need for energy under stress acclimation also corresponds with a degradation of energy-rich storage compounds such as polysaccharides (starch) and storage proteins. A decrease in enzymes related to carbohydrate anabolism (sucrose synthase 1 yielding UDP-glucose) have been found under cold [62]. A decrease in several storage proteins, e.g., legumin-like, 11S seed storage proteins, *etc.*, were also found [62,75]. In contrast, salinity led to an increase in β-conglycinin, a major storage protein in soybean seeds, in young soybean seedling plants indicating a reduced seedling growth under stress with respect to control [64].

### 3.3. Resistance Phase

#### 3.3.1. Stress-Protective Proteins

Several stress factors including drought, salinity, but often also cold, frost, and heat, induce cellular dehydration. A decrease in cell water content in plant cells results in a lack of hydration envelopes and an increased risk of an improper protein folding. Protein disulfide isomerase (PDI) catalyzes a reversible cleavage of disulfide bonds and thus affects protein conformation. Alterations in PDI abundance were found in several studies [37,38,75,88,105]. An increased accumulation of several hydrophilic proteins from COR/LEA family including LEA-II dehydrins [106,107,108,109] and LEA-III proteins such as chloroplast-located COR14b protein [110,111,112] and others [101] was found under cold and drought. Other proteins with chaperone functions include heat shock cognate proteins (HSC) and proteins from HSP superfamily encompassing five families of HSP proteins, HSP110, HSP90, HSP70, HSP60, and small HSP (sHSP) proteins. An increase in several small HSP proteins, but also HSP82 from HSP90 family was found in wheat grain endosperm in developing wheat grains subjected to a heat period [113,114]. An increase in HSP70 and HSC70 was found in watermelon exposed to drought and in tomato exposed to waterlogging, respectively [115,116]. Not only an increase, but also a decrease in some HSP proteins was found in several proteomic studies, e.g., a decrease in HSP70 in drought-treated soybean roots [74] and salt-treated tobacco chloroplast stroma [92], and a decrease in HSP90 in cold-treated winter wheats [75]. An opposite pattern of changes was reported for several sHSP26 protein isoforms in drought-stressed maize leaves [117]. Other proteins with chaperone functions found in proteomic studies include copper chaperone [75,89,118], cystatin [62,119] (cysteine protease inhibitor), serpins [73,84,120,121] (serine protease inhibitor), Zn-dependent metalloproteases [86], DnaK [60], *etc.* Besides their roles as protein chaperones, an interaction of HSP70 with glutathione-related enzymes such as GPX and GR was reported in animal cells thus indicating a role of HSP70 in regulation of cell redox homeostasis [122].

An enhanced risk of protein damage is also reflected by an increase in several ROS scavenging enzymes including catalases, peroxidases, and enzymes associated with ascorbate-glutathione cycle (ascorbate peroxidase, monodehydroascorbate reductase, dehydroascorbate reductase) alterations of which were found in practically all proteomic studies published. The ROS scavenging enzymes differ not only in their substrate specificity, but also in cellular localization and metal cofactors, e.g., Cu/Zn-SOD (cytosolic), Mn-SOD (mitochondrial) and Fe-SOD (chloroplastic). Glutathione peroxidases (GPX) have cytosolic as well as membrane-associated isoforms and catalyze a reduction of lipid peroxides while lipoxygenases catalyze lipid peroxidation A decrease in two lipoxygenases was found in soybean roots exposed to flooding [123]. Peroxiredoxins and thioredoxins are small nuclear-encoded, but chloroplast-located proteins regulating protein activity by a reversible reduction of cysteine residues to disulphide bonds. In plants, thioredoxins are known to regulate activity of Calvin cycle enzymes thus affecting efficiency of photosynthesis. Alterations in several thioredoxin forms, e.g., thioredoxin H and thioredoxin M, were observed under stress including drought [89], a brief freezing stress [70], and salinity [76,77,124] while alterations in peroxiredoxins were found in drought-treated sugar beet [82], wheat [89], salt-treated grapevine [66], maize [49], and cold-treated wheat [69]. A precise regulation of ROS production plays an important role in crop responses to several stresses, namely drought [59,82], drought-induced senescence [89], salinity [77], and heavy metal stress [31,34,37]. Whereas most stress factors led to an increase in the levels of ROS-scavenging enzymes due to imbalances in aerobic metabolism and cellular redox status, a decrease in Cu/Zn-SOD levels was found in soybean roots exposed to waterlogging due to hypoxia [123].

Hypoxia stimulates anaerobic metabolism such as glycolysis and fermentation processes, but also biosynthesis of efficient O_2_ captures such as hemoglobin. In waterlogged maize root cells, an enhanced abundance of coproporphyrinogen III oxidase which catalyzes oxidative decarboxylation of coproporphyrinogen III to protoporphyrinogen IX in heme, a crucial step in hemoglobin biosynthesis, was found by Yu *et al.* [100]. Hypoxia also results in decreased soil pH. Maintenance of stable cellular pH necessary for a proper enzyme function is achieved by an enhanced accumulation of cytoplasmic enzymes with buffering capacity such as glutamate decarboxylase (GDC), malate dehydrogenase (MDH), and NADP-malic enzyme (NADP-ME) [100]. An enhanced abundance of GDC, NADP-ME3, and NADP-ME4 was found in waterlogging-tolerant maize line with respect to the sensitive one [100]. Glutathione S-transferases (GST) represent a large family of enzymes catalyzing glutathione (GSH) conjugation to various substrates. They encompass several structural classes marked by Greek letters φ, τ, ζ, θ. They are known as enzymes involved in detoxification of heavy metals and xenobiotics including several herbicides; however, their glutathionylating activity could also play an important role in regulation of protein activity (*S*-glutathionylation as a posttranslational modification) and in secondary metabolism [125] (*S*-glutathionylated intermediates in metabolism of terpenes, glucosinolates, thiophenes, alliins, *etc.*). Increased GST levels were found under several stresses including wheat exposed to enhanced copper levels [37], cold [69,75], drought [88,94,96,105], salinity [77,81], and cadmium [31].

Pathogenesis-related proteins (PR) encompass a diverse group of 17 protein families (PR2—β-1,3-glucanases; PR3,4,8,11—chitinases; PR5—thaumatin-like; PR6—proteinase inhibitor; PR7—endoproteinase; PR9 -peroxidase; PR10—ribonuclease-like; PR12—defensin; PR13—thionin; PR14—lipid-transfer protein; PR15—germins; PR16—germin-like proteins; PR17—unknown function) involved not only in plant protection against pathogen attack, but also in response to abiotic stresses [126]. Several of them reveal glucanase and chitinase activities aimed at cleavage of fungal cell walls while others reveal ROS scavenging activities (peroxidases, some germins and germin-like proteins) or RNase activities (PR10). Alterations in several PR proteins including β-1,3-glucanases [39], thaumatin-like protein [39,62], PR10 [41,66,127,128], TSI-1 protein [93,124], germin and germin-like proteins [62,77,123,129], PR17 [130], and other protective proteins such as lipid transfer proteins [129], and lipocalins [32] were reported under a wide range of abiotic stresses including drought, salinity, cold, and waterlogging. Plant stress acclimation response is associated with a biosynthesis of several specific stress-protective compounds. For example, changes in enzymes involved in flavonoid and isoflavonoid metabolism participating on biosynthesis of several protective compounds such as anthocyanins and phytoalexins were found not only in pathogen-treated plants, but also in pea plants subjected to salinity [41]. An increased level of chalcone synthase (CHS), a crucial enzyme in flavonoid/isoflavonoid biosynthesis pathway, also interacting with methyl jasmonate and salicylic acid (SA) signaling [131], was found in a drought-sensitive sunflower genotype under dehydration [33].

#### 3.3.2. Structural Proteins

Stress also profoundly affects cellular transport and cytoskeleton. An active ion transport in an opposite direction to physicochemical gradients requires energy. An acquisition of stress tolerance in response to salinity or a hyperosmotic stress (PEG treatment) is associated with an enhanced activity of several Na^+^ transporters and H^+^ transporters involved in Na^+^ exclusion or intracellular compartmentation to vacuole coupled with ATP cleavage [50,56,81,124] (V-ATPase, H^+^-PPase) which is often associated with an increased abundance of ATP synthases components. Severe dehydration also affects water transport via aquaporins resulting in an enhanced abundance of aquaporin proteins as well as aquaporin differential phosphorylation in common wheat exposed to severe osmotic stress [51]. Annexins are cytosolic monomers which can form integral membrane oligomeric complexes enabling transport of several cations including Ca^2+^ and thus involved in cytoplasmic calcium signaling including MAPK kinase cascade and phosphatidylinositol bisphosphate signaling. An increase in annexin level was found in salt-treated potato [93], soybean [95], and tomato plants [124] and a transient elevation was found in drought-treated barley [83] indicating an important role of annexin in abiotic stress signaling process. Voltage-dependent anion channel (VDAC) is a protein complex located in outer mitochondrial membrane which is important for metabolite transport between mitochondria and cytoplasm and which was found increased under several stresses including drought [132], salinity [98], and others. ABC transporters are known to participate on transport of glutathione conjugates into vacuoles and were found elevated under Cd stress [31]. Enhanced actin and β-tubulin levels were found in chicory roots exposed to cold [40], while a decrease in α- and β-tubulin was found in drought-stressed sunflower roots and copper-stressed wheat roots, respectively [33,37].

Stress also reveals profound impacts on plant cell walls. Stress leads to a decreased rate of plant growth and cell division, which also affects cell wall composition. Several proteomic studies dealing with water stress (drought) suggest an increased cell wall lignification, which is reflected by an increased abundance of enzymes involved in lignin biosynthesis such as caffeic acid 3-*O*-methyltransferase (COMT), caffeoyl-coenzyme A *O*-methyltransferase (CCOMT) and phenylalanine ammonia lyase (PAL) [56,74,133,134]. In addition, alterations in enzymes involved in metabolism of cell wall polysaccharides such as cellulose, hemicelluloses, and pectins were found; for example, UDP-glucuronic acid decarboxylase, β-d-glucan exohydrolase, UDP-glucose pyrophosphorylase in salt-treated barley [72], xyloglucan *endo*-transglycosylase and UDP-glucosyl transferase BX9 in drought-, salt- and waterlogging-treated maize roots [49,100,135]. These data indicate a substantial cell wall remodeling in response to stress usually leading to a decreased elasticity due to cell elongation cessation and an increased lignification as a potential barrier against dehydration stress. In contrast, a decreased lignification and increased cell wall loosening were found under waterlogging [78,100]. Decreased levels of β-1,3-glucanases, β-glucosidases, and methionine synthase as a precursor of SAM, a methyl donor for monolignol synthesis, indicate an inhibitory effect of waterlogging on wheat seedling growth [136]. This phenomenon may be associated with decreased ROS and jasmonate levels under flooding [123].

### 3.4. Comparison of Various Abiotic Stresses, Stress Recovery

Regarding crop stress acclimation responses, plant stress responses include both common as well as specific features. Common features of several abiotic stresses studied (cold, frost, drought, salinity) include cell dehydration, *i.e.*, dehydrative stress, and imbalances in aerobic metabolism, *i.e.*, oxidative stress. Cell dehydration leads to an enhanced biosynthesis of low-molecular osmolytes and hydrophilic proteins as well as chaperones to prevent protein misfolding and aggregation. Imbalances between chloroplast and mitochondrial electron transport chains and enzymatic reactions (Krebs cycle, Calvin cycle) resulting in an enhanced ROS formation lead to an enhanced accumulation of ROS scavenging enzymes. However, several stress factors also reveal specific effects on plant cell structure and metabolism. Heat is associated with an enhanced risk of protein misfolding leading to enhanced levels of HSPs, especially sHSPs. Salinity can be characterized by a specific ionic effect, *i.e.*, penetration of Na^+^ into cell cytoplasm, which leads to an activation of ATP-dependent ion channels resulting in Na^+^ exclusion or intracellular compartmentation (vacuolar sequestration). Specific effects of waterlogging include hypoxia, resulting in an activation of anaerobic metabolism (glycolysis, fermentation), and a decreased soil pH resulting in an enhanced abundance of several cytosolic enzymes with buffering capacity such as NADP-ME, MDH, and others. Imbalances in metal nutrients as well as heavy metal stress lead to an enhanced abundance of several proteins with ion chelating functions (ferritin, phytochelatins) as well as ROS scavenging enzymes since free metal ions act as efficient ROS catalyzers (Table 1).

Practically all proteomic studies dealing with crop response to an abiotic stress are focused on plant stress acclimation. However, it should be kept in mind that recovery after a cessation of stress stimulus is equally important since it profoundly affects further plant growth and development. However, recovery after a stress treatment is still being seldom studied in crops. An exception represent a paper on drought-treated wheat cultivars with differential drought tolerance where plant proteome response at one day after rewatering was studied [132] as well as a paper on drought-treated soybean followed by four days of recovery [74]. In wheat, proteome analysis of a rewatering response has revealed an increased abundance of 8 out of 12 glycolysis enzymes in tolerant cultivar Excalibur indicating an enhanced need on energy during a recovery treatment. HCF136 which is a protein involved in repair and assembly of OEC and PSII complexes significantly increased in tolerant Excalibur under rewatering indicating a quick PSII repair after stress cessation. In contrast, dehydration-induced proteins COR410 and SDi-6 revealed a significant decrease at rewatering indicating a cessation of the adverse impacts of a stress treatment. In soybean, rewatering led to an increase in some regulatory proteins potentially involved in the delay of senescence and PCD (eIF5A, MADS-box TF KIP, pentatricopeptide repeat protein) which were downregulated upon drought stress. In common bean, recovery after a long-chilling stress (10 °C per 16 days) as well as short-chilling stress (10 °C per 24 h) was studied by Badowiec and Weidner [137]. Recovery after chilling stress led to a decrease in several stress-related proteins such as LEA1, HSP, GST, SAMS, while an increase in proteins involved in energy metabolism (mtATP synthase CF1α,β; pyruvate kinase) indicating an enhanced need for energy to achieve novel homeostasis.

**Table 1:** Basic characteristics of the major effects of abiotic stresses on plant biological mechanisms including plant stress response. Abbreviations: COR—cold-regulated (protein); GDC—glycine decarboxylase; HSP—heat shock (protein); LEA—late embryogenesis-abundant (protein); MDH—malate dehydrogenase; NADP-ME—NADP malic enzyme; PCD—programmed cell death; ROS—reactive oxygen species; XET—xyloglucan *endo*-transglycosylase.

Stress Factor	Stress Effect	Plant Response
Low temperature (cold, frost)	Imbalance between water uptake and water release—cellular dehydration. Imbalance between non-enzymatic electrontransport reactions and enzymatic reactions (Krebs cycle, Calvin cycle) in chloroplasts and mitochondria—oxidative stress (enhanced ROS formation)	Enhanced biosynthesis of low-molecular osmolytes (proline, sugars, betaines) and hydrophilic proteins (COR/LEA) Enhanced biosynthesis of ROS scavenging enzymes, downregulation of crucial photosynthetic enzymes
Heat	Enhanced risk of protein misfolding	Enhanced accumulation of HSPs, especially sHSPs
Drought	Imbalance between water uptake and water release—cellular dehydration Imbalance between non-enzymatic electron transport reactions and enzymatic reactions (Krebs cycle, Calvin cycle) in chloroplasts and mitochondria—oxidative stress Reduced growth	Enhanced biosynthesis of low-molecular osmolytes (proline, sugars, betaines) and hydrophilic proteins (COR/LEA) Enhanced biosynthesis of ROS scavenging enzymes, downregulation of crucial photosynthetic enzymes Enhanced cell wall lignification
Salinity	Decreased soil water potential—cellular dehydration—osmotic effect Enhanced Na^+^ penetration—ionic effect	Enhanced biosynthesis of low-molecular osmolytes (proline, sugars, betaines) and hydrophilic proteins (COR/LEA) Enhanced levels of ATP-dependent Na^+^/H^+^ transporters resulting in Na^+^ exclusion (plasma membrane) or Na^+^ intracellular compartmentation (tonoplast)
Nutrient deficiencies and heavy metal stress	Enhanced metal ion penetration—oxidative stress	Enhanced levels of metal-chelating proteins (ferritin, phytochelatins, LEA) and pathways involved in their biosynthesis (Yang cycle); enhanced ROS scavenging enzymes

## 4. Differences in Stress Response between Tolerant and Sensitive Genotypes

Most temperate crop plants represent plant species with a relatively large and diverse genetic pool despite an intensive selection during a breeding process. Although modern cultivars are bred primarily for crop quality and high yield, there are also several landraces and wild relatives adapted to harsh environments. Moreover, modern breeding for high-yielding cultivars in harsh environments such as dry environments in Australia or freezing temperatures in Canada, have also led to a release of tolerant cultivars. In addition, several landraces, wild accessions and relative species such as *Hordeum marinum*, *Solanum commersoni*, *Thinopyrum ponticum*, *etc.*, represent genetic materials adapted to harsh environments, which can be employed as potential genetic resources of alleles underlying an enhanced stress tolerance.

Transcriptomic and proteomic studies focused on comparison of plant genotypes or related species with contrasting stress tolerance, e.g., *Arabidopsis thaliana* (glycophyte) and *Thellungiella salsuginea* (halophyte), have revealed increased levels of stress-inducible proteins in tolerant genotypes even in the absence of stress [138]. Tolerant plants are thus able to efficiently diminish adverse effects of stress when compared to sensitive plants. Tolerant plants reveal constitutively enhanced levels of several stress-responsive proteins including transcriptional regulators [59] (e.g., SWIB/MDM2 protein, Myb protein, B-Peru-like protein involved in anthocyanin biosynthesis) and thus also several stress-protective proteins, ROS scavenging enzymes and proteins involved in metabolism of stress-related phytohormones. For example, constitutively enhanced levels of r40c1 protein which belongs to a class of ABA-induced proteins, and an enhanced level of lipoxygenase (LOX), an enzyme involved in biosynthesis of jasmonic acid, and increased levels of several chaperones (HSP70, HSP90, CPN60-α,β, cyclophilin A), S-adenosylmethionine synthase (SAMS), glutathione-*S*-transferase (GST), *etc.*, were found in drought-tolerant barley Basrah with respect to drought-sensitive Golden Promise [59] as well as in drought-tolerant wheat Nesser with respect to drought-sensitive Opata [57]. A comparative proteomic study of common wheat (*Triticum aestivum*) and its hybrid *Triticum aestivum* × *Thinopyrum ponticum* exposed to salinity has revealed an increased abundance of V-ATPase involved in Na^+^ vacuolar compartmentation [54]. Increased levels of ascorbate peroxidase (APX) and catalase (CAT) were found in drought-tolerant maize genotype with respect to the drought-sensitive one [117]. Increased levels of Mn-SOD as well as other mitochondrial redox enzymes and protective proteins were found in salt-tolerant wheat × *Lophopyrum elongatum* amphiploid with respect to salt-sensitive common wheat cv. Chinese Spring as well as in cold-tolerant winter wheat with respect to the less tolerant one, respectively [97,99]. An increased level of cold-inducible dehydrin proteins WCS120 in common wheat and DHN5 in barley in frost-tolerant winter cultivars with respect to less tolerant winter genotypes as well as spring ones was found not only upon cold, but also at mild cold to optimum growth temperatures [63,139]. Moreover, a differential PTM such as a differential phosphorylation level of YxSKn-type dehydrin DHN5 was reported in differentially drought-tolerant genotypes of durum wheat [107]. Consistent with constitutively increased levels of several stress-protective proteins in tolerant genotypes, a higher stress-inducible increase of some stress-responsive proteins such as HSP70 and thioredoxin H was found in sensitive genotypes with respect to tolerant ones when exposed to a stress treatment [124]. Due to a constitutively increased abundance of several stress-protective proteins in stress-tolerant genotypes with respect to stress-sensitive ones, tolerant genotypes are able to fulfill the demands on enhanced energy during the stress acclimation process. This fact is reflected by enhanced levels of photosynthesis-related proteins (OEC components; carbonic anhydrase; RubisCO large subunit) and ATP biosynthesis (ATP synthase β subunit) found in tolerant genotypes with respect to sensitive ones [94,96,120,130,140]. Consistent with this hypothesis, a relatively lower decrease in energy-rich storage compounds such as storage proteins (legumin-like protein, 11S seed storage protein) was found in tolerant winter wheats than in less tolerant winter genotypes and sensitive spring genotypes upon cold treatment [62,75].

A lower damage of plant tissues and lower energy costs on stress acclimation in tolerant genotypes in comparison to sensitive ones could also result in a relatively more positive effect on novel protein biosynthesis, plant growth, and development in tolerant genotypes compared to sensitive ones when subjected to stress. An increase in eIF3 and mitochondrial EF-TuM was found in drought-tolerant maize genotype CE704 subjected to six days of dehydration while a decrease in eEF1D was found in drought-sensitive maize genotype 2023 under the same conditions [117]. A relatively lower decrease in eIF5A3 factor regulating not only protein biosynthesis, but also cell cycle (cell division) was found in *Triticum aestivum* × *Thinopyrum ponticum* hybrid Shanrong 3 with respect to its parental wheat cultivar Jinan 177 under salinity [54]. Consistent with a factor regulating cell division, a relatively higher level of DWARF3, a protein involved in gibberellin biosynthesis, was found in salt-tolerant hybrid with respect to its parent under salt stress indicating a relatively higher rate of cell division and plant development in the tolerant genotype [47,54]. Consistent with a relatively lower growth inhibition in tolerant cultivars vs sensitive ones under stress, a relatively increased level of enzymes involved in cell wall elongation such as xyloglucan *endo*-transglycosylase (XET) was found in drought-tolerant grapevine cultivar with respect to drought-sensitive one [66]. In addition, novel protein biosynthesis may be not impaired to such an extent in tolerant cultivars with respect to sensitive ones as shown by a significant decrease of chloroplast 30S ribosomal protein S10, a protein crucial for binding of tRNA to ribosomal surface and initiation of protein biosynthesis, in salt-sensitive canola cultivar Sarigol compared to no significant change in salt-tolerant cultivar Hyola 308 when exposed to salt stress [67].

## 5. Combinations of Multiple Stress Factors

In nature, plants are usually exposed to multiple abiotic (and biotic) stresses [141,142]. However, plant stress responses to combined stress treatments are seldom studied. A few proteomic studies dealing with combined stress treatments have shown that plant response to a combined stress treatment is specific when compared to the individual stress factors applied separately. Since no simple assumptions on an additive effect of individual stress treatments can be applied, plant response to combined stress treatments deserves to be studied.

Peng *et al.* [47] compared the effects of drought and salinity as two separate treatments applied on a relatively sensitive bread wheat cv. Jinan 177 and its somatic hybrid with tall wheatgrass *Thinopyrum ponticum* named Shanrong 3. Comparison of proteome response to both treatments has revealed that salinity induced significant alterations in a higher number of proteins than drought as a consequence of an ionic effect of salinity stress.

Rollins *et al.* [91] studied the effects of drought (15% soil water content), heat (36 °C) and a combined drought and heat treatment in two relatively drought-tolerant barley genotypes originating from differential drought environments, Syrian landrace Arta and Australian cultivar Keel, differing in their drought response strategies. Drought led to a reduction in plant growth while maintaining relatively stable proteome composition. In contrast, heat led to enhanced protein damage, especially of PSII components, and thus an enhanced need for energy due to an increased protein turnover. An enhanced abundance of ROS scavenging enzymes and protective proteins (HSPs, a thermostable RubisCO activase B isoform) was found in heat-treated plants indicating an imbalance between the rates of primary (light-dependent) and secondary (light-independent) photosynthetic reactions and an enhanced risk of protein damage under heat stress.

Li *et al.* [143] studied the effect of a spring freezing in combination with either drought or waterlogging (water stresses) on winter wheat cv. Yannong leaves sampled from plants in anther connective tissue formation stage. Differences between the individual treatments and combined treatments were observed. For example, HSP70 decreased in response to a single freeze stress treatment while the same protein increased in response to combined stress treatments. In contrast, decreased levels of chloroplast ATP synthase β subunit and mitochondrial ATP synthase α subunit in both single freezing and combined freezing and waterlogging treatments are consistent with a decrease in Ca^2+^ and Mg^2+^-ATPase activities and an observed damage of PSII under waterlogging stress.

Yang *et al.* [144] investigated an effect of drought and heat (32 °C) treatment on proteome composition of wheat grain in the stage of terminal spikelet and anthesis. Several proteins revealed a specific response to each stress treatment while only a few common proteins involved in redox metabolism, defense, carbohydrate metabolism, and storage revealed an analogous response to multiple stress treatments. Proteins responding exclusively to heat stress include increased cinnamoyl-CoA reductase, TCTP (translationally-controlled tumor protein), cell division control protein, and heat shock cognate 70 (HSC70) and a decreased 14-3-3 protein.

A comparison of contrasting water stresses—drought and flooding—was carried out by Oh and Komatsu [78] in soybean seedlings. The results have shown differentially regulated stress responses—an increase in enzymes involved in regulation of redox homeostasis was found in drought-stressed plants while an increase in anaerobic metabolism-related (fermentation) enzymes was found in flooded plants. Moreover, an opposite pattern of changes in SAM synthetase (SAMS) was found in drought-treated plants vs flooded ones indicating an increase in SAMS under drought while a decrease in SAMS under flooding. Since SAM is a universal cell methylation agent involved in lignin biosynthesis, the differential pattern of SAMS may be related to changes observed in root cell wall lignification in soybean seedlings under the two treatments corresponding to an increased cell wall lignification under drought while a decreased cell wall lignification under flooding, respectively.

Effects of drought, cold, and herbicide paraquat treatments on pea mitochondrial proteome were compared by Taylor *et al.* [42]. The strongest adverse effects on mitochondrial proteins resulting in an oxidative damage were observed under paraquat treatment, followed by chilling while drought revealed the mildest effects. Mitochondria isolated from stressed pea plants maintained their electron transport chain activity; complexes of mitochondrial electron transport chain were least damaged by oxidative stress, F_1_F_o_ ATP synthase complex was more damaged while enzymes of carbon metabolism in mitochondrial matrix were significantly modified by oxidation. Moreover, increased lipid peroxidation and a decrease in inner membrane import proteins were observed. Differential changes in abundance of several HSP proteins with an induction of HSP22 and opposite patterns in several isoforms of HSP70 were also found under both chilling and drought treatments.

## 6. Conclusions and Future Perspectives

During the past two decades, a boom of high-throughput separation techniques together with whole genome sequencing projects have enabled the development of “Omics” approaches in the study of plant response to environmental cues at transcript, protein, and metabolite levels. Proteome, a whole of proteins present in a given tissue at a given time, represents an important component of plant response to environment since proteins are directly involved in constituting the resulting plant phenotype. Recent publications of complete genome sequence in major crops have enabled the researchers an identification of practically any novel protein detected in a proteomic analysis. However, large genomes of several crops, especially the allohexaploid genome of common wheat (*T. aestivum*), are poorly annotated containing several draft sentences and genes of unknown functions. Moreover, unlike the one genome, an infinite number of proteomes can be described for a given organism depending on tissue type, developmental stage, and environment. Moreover, a given protein can adopt multiple forms differing in their pI and MW values on 2DE gels as a result of several posttranscriptional and posttranslational protein modifications. Therefore, one gene can encode multiple different proteins.

Currently, proteomic studies dealing with stress treatments in crop plants are still dominated by comparative studies focused on total proteome, *i.e.*, comparisons of proteomes in control vs stress-treated plants as well as genotypes with differential responses to a given stress. However, it can be suggested that in the future, proteomic studies will become more specific and focused on a response of a defined tissue or cell line compared to plant organs dominating in current studies due to employment of laser microdissection and other isolation techniques. Moreover, subcellular proteomics will become more common due to improved cell fractionation techniques. Specific protein isoforms and posttranslational modifications associated with characterization of their individual roles in plant response to environmental cues will become increasingly studied since current proteomic studies clearly show that different protein isoforms can reveal a differential response to the same environmental cue. Moreover, study of protein-protein interactions will also become an inevitable part of proteomic research since protein interactions are crucial for a final plant cell response. As an example of an interactomics study applied in crop stress response, a paper of Tardif *et al.* [145] on interaction networks of signaling proteins (Ran-related GTP binding protein, phospholipase C) and transcription factors involved in vernalization regulation (TaVRT1/VRN1, TaVRT2, VRN2, TaFT) in winter wheat studied by classical yeast-two-hybrid approach and validated *in planta* by split-GFP technique can be given.

Abiotic stress factors belong to the main environmental factors affecting crop growth and productivity. The major crops of a temperate climate zone including common and durum wheat, barley, maize, and soybean are grown worldwide in a very diverse environments (including semi-arid and arid areas of Australia and Middle East, temperate climate areas with harsh winter conditions and a high risk of freezing damage, areas endangered by soil salinity, imbalances in mineral nutrition, soil pollution by heavy metals, and other factors). Moreover, in nature, plants usually have to cope with combinations of several diverse stress factors. Proteomic research aimed at understanding crop responses to abiotic stresses is still in its beginnings despite more than a decade of high-throughput proteomic experiments. One way to use proteomic outputs to be more beneficial e.g. for breeders is to aim studies for detailed characterization of tolerant to sensitive cultivars of different crops and relate these data to particular environment and management systems. The major reasons include a unique response of different plant organs and tissues as well as growth stages, and different stress treatments including stress dynamics. It has also been proven that combined stress treatments induce a unique plant stress response at the proteomic level, which could not be described as a simple additive effect of the individual stress treatments. Therefore, combined stress treatments also need to be studied due to their frequent occurrence in natural conditions. As an example, combined heat and drought stress occurrence, but also freezing in combination with either drought or waterlogging can be given.

**Figure 2:**
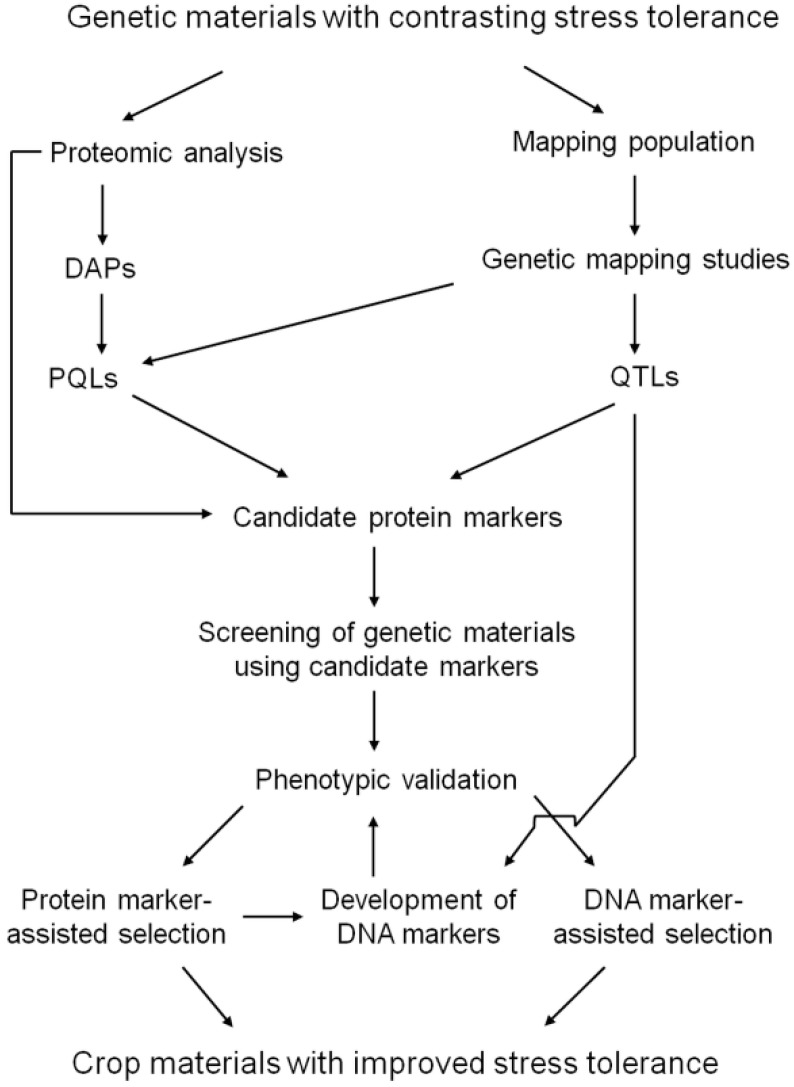
A schematic workflow of the utilization of candidate protein markers in breeding for an improved crop stress tolerance. Abbreviations: DAPs—differentially abundant proteins; PQL—protein quantitative loci; QTL—quantitative trait loci.

Proteomic analyses can lead to an identification of proteins revealing common response to multiple stress treatments as well as proteins responding only to specific stress conditions. Both types of proteins can represent potential candidates for testing new plant materials for their potential stress tolerance during prescreening procedures in crop breeding programs aimed at an improvement of crop stress tolerance. According to Riccardi *et al.* [119], a protein considered a potential stress marker candidate has to fulfill the following two criteria: it has to be induced by a given stress factor and its protein quantitative locus (PQL) has to co-localize with a corresponding quantitative trait locus (QTL) for a given trait associated with stress tolerance. Proteomics of crop response to abiotic stresses has thus a large potential in crop breeding due to its large potential in designing novel breeding materials with specific characteristics (Figure 2). Application of the results of proteomic analyses can lead to selection of key protein markers including specific protein isoforms or PTMs, respectively, for a given crop feature which will be then tested in large sets of breeding materials as a part of routine procedures during the breeding selection. As an example, our results obtained on cold-responsive dehydrin proteins WCS120 in common wheat and DHN5 in barley as potential markers of plant acquired frost tolerance can be given [63,108,109,139]. It can be concluded that in the future, analysis of potential protein markers for some desired trait will probably become a routine part of a modern breeding process.

## Supplementary Materials

Supplementary materials can be found at http://www.mdpi.com/1422-0067/16/09/20913/s1.

## Abbreviations

2Cys-Prx: 2-cysteine peroxiredoxin
2DE: two-dimensional electrophoresis
2D-DIGE: two-dimensional differential in-gel electrophoresis
β-CAS: β-cyanoalanine synthase
ABA: abscisic acid
ACP: acyl carrier protein
ADH: alcohol dehydrogenase
AGPase: ADP glucose pyrophosphorylase
AOX: alternative oxidase
APX: ascorbate peroxidase
AQP: aquaporin
AsA: ascorbic acid
bHLH: basic helix-loop-helix (protein)
BN-PAGE: blue-native polyacrylamide gel electrophoresis
CaM: calmodulin
CCOMT: caffeoyl-coenzyme A *O*-methyltransferase
CHS: chalcone synthase
COMT: caffeic acid *O*-methyltransferase
COR: Cold-regulated (protein)
CPN: chaperonin
CS: cysteine synthase
CDPK: calcium-dependent protein kinase
CHS: chalcone synthase
DAP: differentially abundant proteins
DH: double haploid (line)
DHAR: dehydroascorbate reductase
DON: deoxynivalenol
ENO: enolase
ESI: electronspray ionization
FBP ALDO: fructose-1,6-bisphosphate aldolase
FRK: fructokinase
FWC: field water capacity
GAPDH: glyceraldehyde-3-phosphate dehydrogenase
GAPDH B: glyceraldehyde-3-phosphate dehydrogenase B form
GDC: glycine decarboxylase
GDH: glutamate dehydrogenase
GLP: germin-like protein
GPX: glutathione peroxidase
GRP: glycine-rich protein
GS: glutamine synthetase
GST: glutathione S-transferase
HPLC: high performance liquid chromatography
Hsc: heat shock cognate protein
IFR: isoflavone reductase
iTRAQ: isobaric tag for relative and absolute quantification
LC: liquid chromatography
LEA: Late embryogenesis-abundant (protein)
LOX: lipoxygenase
LTP: lipid transfer protein
LTQ-FTICR: linear quadruple trap-Fourier transform ion cyclotron resonance
MALDI-TOF/TOF: matrix-assisted laser desorption ionization time-of-flight/time-of-flight (spectrometry)
MAPK: mitogen-activated protein kinase
MDAR: monodehydroascorbate reductase
MDH: malate dehydrogenase
MIPS: *myo*-inositol-1-phosphate synthase
MS: mass spectrometry
MSSP2: monosaccharide sensing protein 2
NADP-ME: NADP malic enzyme
NBS-LRR: nucleotide-binding site leucine-rich repeat protein
NEPHGE: non-equilibrium pH gel electrophoresis
NDPK: nucleoside diphosphate kinase
NIL: near-isogenic line
OEE: oxygen evolving enhancer (protein)
PBS: phosphate buffer saline
PC: plastocyanin
PDI: protein disulfide isomerase
PDX: pyridoxal biosynthesis protein
PEG: polyethylene glycol
PGK: phosphoglycerokinase
PGM: phosphoglyceromutase
POX: peroxidase
PPase: inorganic pyrophosphatase
PPDK: pyruvate phosphate dikinase
PPR: pentatricopeptide repeat (protein)
PRK: phosphoribulokinase
Prx: peroxiredoxin
PS: photosystem
PVP: polyvinyl pyrrolidone
qTOF: quadrupole time-of-flight
RubisCO: ribulose-1,5-bisphosphate carboxylase/oxygenase
RubisCO LSU: RubisCO large subunit
RubisCO SSU: RubisCO small subunit
RWC: relative water content
S: sensitive (genotype)
SA: salicylic acid
SAMS: S-adenosylmethionine synthase
SBP: sedoheptulose-1,7-bisphosphatase
SHMT: serine hydroxymethyltransferase
SnRK: sucrose non-fermenting-related protein kinase
SOD: superoxide dismutase
SUS1: sucrose synthase 1
SWC: soil water content
T: tolerant (genotype)
t: genotype less tolerant than T
TCA: trichloroacetic acid
TCTP: translationally controlled tumour protein
TF: transcription factor
TLP: thaumatin-like protein
TPI: triose phosphate isomerase
Trx: thioredoxin
TSI-1: tomato salt-induced 1 (protein)
V-ATPase: vacuolar ATPase
VDAC: voltage-dependent anion channel
WCS: Wheat Cold-specific (protein)
WRAB: Wheat responsive-to-ABA (protein)
XET: xyloglucan *endo*-transglycosylase
Y2H: yeast-two-hybrid (screen)
