# Supplementary material for: Biological Networks Underlying Abiotic Stress Tolerance in Temperate Crops—A Proteomic Perspective
Source: Int J Mol Sci. 2015 Sep 1;16(9):20913–42. doi: 10.3390/ijms160920913 (PMC4613235; doi:10.3390/ijms160920913)
Supplement: Supplementary file 1 [file ijms-16-20913-s001.pdf]

Supplementary Information

Table S1. A list of proteomic studies focused on abiotic stress responses in selected temperate crops.

Plant Material	Treatment	Methods	Major Differentially-Abundant Proteins (DAP)	Reference
**Low temperature (cold, frost)**				
Winter wheats (*Triticum aestivum*) Mironovskaya 808 (T) and Bezostaya 1(t)—leaf	2 °C (21 days)	0.1 M Tris-HCl, pH 9; 2DE LC-MS/MS	Up: WCS120, WCS19, COR14a—higher levels in T	[109]
Winter wheats Norstar (T) and Azar2 (t)—leaf	2 °C (0, 14, 28, 42, 56 days)	2DE MALDI-TOF/TOF	Up: COR/LEA (WCOR14a, WRAB17, WRAB18); Cu/Zn-SOD, 2-2-Cys Prx, GST—higher levels in T	[69]
Winter wheat Cheyenne (T)—leaf	4 °C (63 days)	TCA/acetone; 2DE MS/MS	Up: WCOR18, WRAB17, WCOR615; VER2, glycine-rich RNA binding protein	[61]
Winter wheat cv. Norstar (T)—crown	4 °C (1, 23, 53 days)	Y2H (*Saccharomyces cerevisiae*); split GFP (*Nicotiana benthamiana*)	Interaction partners: TaVRT-1/VRN-1, TaVRT-2, VRN-2, TaAP2, TaHD, TaFT (vernalization and development regulation); RanGTP, phospholipase C, receptor-like protein kinase (stress signaling)	[145]
Spring wheat Kohdasht (S)—leaf	4 °C (42 days) Control: 20 °C (42 days)	2DE nanoLC-MS/MS	Up: APX, DHAR, COR/LEA, cysteine proteinase, proteasome subunit α Down: glycolysis (GAPDH, TPI), Krebs cycle enzymes (MDH), ATP synthase β,ε; PSII subunits	[60]
Winter wheats Mironovskaya 808 (T) and Bezostaya 1 (t)—crown	6 °C (0, 3, 21, 84 days)	TCA/acetone/phenol; 2D-DIGE MALDI-TOF/TOF	298 DAP (202 identified) Up: 3-PGK, TPI, PGM, ENO; HSP70; MDAR, DHAR, GPX, GST Down: ALDO, GAPDH; SUS1, UDP-glucose pyrophosphorylase; 14-3-3; HSP90; APX Vernalization: Chopper chaperone Genotypic differences: MDH, legumin-like protein—higher in T than t	[75]
Wheat cv. Yumai 34—leaf	−5 °C (3 days)	2DE MALDI-TOF/TOF	75 DAP—Up: 52 (COR/LEA, APX, Cu/Zn-SOD, Trx M) Down: 18 (RubisCO LSU, SSU)	[70]
Winter wheat Samanta (T), spring wheat Sandra (S)—crown	4 °C (0, 3, 21 days)	TCA/acetone/ phenol; 2D-DIGE MALDI-TOF/TOF	58 DAP (36 identified) Up: GAPDH, β subunit ATP synthase, CPN60-α, CPN60-β Down: FRK-2, SUS1, 11S seed storage protein Genotypic differences: methionine synthase, eIF3, eIF5A2—higher in T; VER2, sGRP—higher in S	[62]

Table S1. *Cont.*

Plant Material	Treatment	Methods	Major Differentially-Abundant Proteins (DAP)	Reference
Winter wheats—Shixin 828 (T), Shiluan 02-1 (t)—leaf	−8 °C (5 h)	TCA/acetone; 2DE MALDI-TOF/TOF	RubisCO LSU and SSU; α and β subunit ATP synthase; V-ATPase; MDH; Genotypic differences: RubisCO LSU and SSU, PRK; Mn-SOD—higher in T than t	[97]
Wild wheat (*Triticum urartu*)—leaf	4 to 6 °C (28 days) followed by −2 °C (12 h)	TCA/acetone; 2DE MALDI-TOF/TOF	34 identified proteins—25 up- and 9 down-regulated Up: LEA-III, WCOR14, PR4; OEE1, chloroplastic ribosomal protein L12 Down: RubisCO SSU	[101]
Barley (*Hordeum vulgare*) Winter barley Luxor (T)—crown, leaf	3 °C (0, 1, 21 days), −3 °C (1 day)	TCA/acetone/phenol; 2D-DIGE MALDI-TOF	Up: HSP70; OEE1 (PsbO), Down: eEF-Tu; GS1 and 2; UDP-glucose 6-dehydrogenase Both leaf and crown: AAA ATPase, V-ATPase; eEF-Tu, CPN60, 60S and 40S ribosomal proteins; GS	[118]
Chicory (*Cichorium intybus*)—root	5 °C (several days)	2DE MALDI-TOF/TOF	619 identified proteins (most abundant: actin, β-7 tubulin, NDPK, Cu/Zn-SOD, TPI)	[40]
Sunflower (*Helianthus annuus*) cvs Hopi (S), PI543006, BSD-2-691 (T)—leaf	15/5 °C (day/night)—7 days	2DE LC-MS/MS, spectral counting	Identified proteins: 718 (Hopi), 675 (PI543006), 769 (BSD-2-691) (lipocalin, Trx, 60S ribosomal protein L10A, L4-2, chloroplast 30S ribosomal protein S4)	[32]
**Heat**				
Common wheat—Fang (T), Wyuna (S)—grain endosperm	40/25 °C (day/night)—15,16,17 days post-anthesis	TCA/acetone 2DE MALDI-TOF; MS/MS Q-TOF	Genotypic differences: Seven small HSP (16.9 kD class I HSP) proteins unique to T	[113]
Common wheat—Thésée—grain	34/10 °C (day/night)—697 and 763 °C-d (degree-days)	Sodium-phosphate buffer; 2 DE MALDI-TOF	42 identified proteins Up: 20 kD sHSP, 17 kD class II HSP; HSP82 (HSP90 family); eEF-Tu, V-ATPase subunit E Down: starch biosynthesis enzymes granule-bound starch synthase, glucose-1-phosphate adenyltransferase; β-amylase; β subunit ATP synthase	[114]

Table S1. *Cont.*

Plant Material	Treatment	Methods	Major Differentially-Abundant Proteins (DAP)	Reference
Alfalfa (*Medicago sativa*) cv. Huaiyin—leaf	40 °C (24, 48, 72 h)	2DE MALDI-TOF/TOF	81 DAP: GS, PDI, peptidyl-prolyl *cis-trans* isomerase, RubisCO activase, sHSPs, IFR; Tic40, Toc75—chloroplast import machinery	[38]
**Drought**				
Common wheat—spring wheats Arvand, Khazar-1, Kelk Afghani—grain	Field conditions (Azarbayjan) plus artificial irrigation	2DE MALDI-TOF/TOF	121 (57 identified) Up: Trx *h*, 1-Cys peroxiredoxin, GST; PDI; LEA, sHSP17, HSP70	[105]
Wheat Iranian landraces N14, N49—stem (senescence stage)	80%–90% FWC (control), 50% FWC (drought)—10, 20, 30 days after anthesis	2DE MALDI-TOF/ TOF; Q-TOF	135 DAP (82 identified)—redox (GST, Prx-5, Trx M), chaperonin, protein metabolism (chloroplast 50S ribosomal protein L4, proteasome regulatory subunit 14, Clp protease)	[89]
Australian wheats Kukri (S), Excalibur, RAC875 (T)—leaf	Water witholding until leaf wilting in Kukri (S)—14, 24 days, and rewatering (25 days)	TCA; nanoLC-MS/MS iTRAQ 8plex	1299 identified proteins Increase in ROS metabolism-associated proteins (CAT, Cu/Zn-SOD, Mn-SOD), decrease in photosynthesis and Calvin cycle-related proteins (RubisCO; PSI subunit VII PsaC) Genotypic differences: COR410—higher increase in T than S	[132]
Wheat spring genotypes Ninchun 4, Chinese Spring—grain	14 days after flowering	2DE MALDI-TOF, MALDI-TOF/TOF	152 DAP (96 identified)—Up: TCTP, Down: APX, RubisCO	[120]
Common wheat cv. Nesser (T), Opata M85 (S)—root	21 °C; 40 % humidity—combined effect of drought and ABA (100 µM)	nanoLC-MS/MS iTRAQ	1656 identified proteins 805 ABA-responsive proteins: LEA, protein phosphatases PP2C; Genotypic differences: HSP70, HSP90; 14-3-3, G-proteins; V-ATPase—higher in T; β-expansin, porins—higher in S	[57]
Wheat cvs. Seri M82 (T), SW89.5193 (S)—leaf, root	80% FWC (control), 20% FWC (drought)	2DE nanoLC-ESI-MS/MS	Up: GST, SOD Genotypic differences: MDH, SAMS—up in T; 14-3-3—down in S	[88]

Table S1. *Cont.*

Plant Material	Treatment	Methods	Major Differentially-Abundant Proteins (DAP)	Reference
Durum wheat cv. Ofanto—leaf	70 % FWC for 7 days (control); 57% FWC for 7 days (stress)	175 mM Tris-HCl, pH 8.8, TCA-acetone; 2DE MALDI-TOF	36 identified proteins Up: carbonic anhydrase, RubisCO LSU Down: RubisCO SSU, Calvin cycle enzymes (ALDO, PRK); ATP synthase CF1 α; plastidic GS2a,b,c	[30]
Durum wheat cv. Kiziltan (S), emmer (*T. dicoccoides*) lines TR39477, TTD22 (T)—leaf	9 days water withholding	2DE nanoLC-ESI-MS/MS	75 identified proteins, 11 candidates for drought tolerance Genotypic differences: TPI, ATP synthase CF1 (efficient carbohydrate metabolism and ATP production)—higher in T; β-1,3-glucanase, β-1,4-glucanase, XET (cell wall remodelling for osmotic adjustment and energy source); methionine synthase—higher in S	[96]
Barley cv. Basrah (T) and Golden Promise (S)—leaf, root	7 days water withholding Control: 80% RWC; Drought: 70% RWC (T), 60% RWC (S)	10 mM PBS, TCA-acetone; 2D-DIGE MALDI-TOF	Identified proteins: 24 (leaf), 45 (root) Up: ABA-induced protein r40c1, small G-protein Rab2, Myb-like protein, 14-3-3 protein Down: GST, GPX Genotypic differences: Enhanced regulation of ROS (APX, CAT, LOX, class III POX) and protein folding in T than in S	[59]
Barley—8 Egyptian accessions, 2 selected for proteome analysis 15141 (T), 15163 (S)—leaf	24 °C; 70% FWC (control); 5 days at 10% FWC (stress)	TCA/acetone; 2D-DIGE MALDI-TOF	Up: PDI, Hsp90, Hsp100 (Clp protease), chloroplastic ATP synthase CF1 α; Genotypic differences: PPDK, Hsp70, zinc metalloprotease—higher in T than S; proteins involved in osmolyte biosynthesis (betaine aldehyde dehydrogenase, methionine synthase, SUS1)—higher in S than T	[86]
Barley cv. Golden Promise—leaf	Ca 100% FWC (control); 25% FWC (stress)—28 days—combined effect of drought and *Piriformospora indica*	TCA/acetone; 2DE MALDI-TOF/TOF	45 identified proteins Up: RubisCO activase A, RubisCO SSU, CCOMT Down: PRK, ACP Effect of *P. indica*: RubisCO SSU, PSI Fe-S, chl *a*/*b* binding protein; CCOMT, APX, 30S ribosomal protein 3, V-ATPase, 2-Cys Prx—higher in inoculated than control plants under stress	[56]

Table S1. *Cont.*

Plant Material	Treatment	Methods	Major Differentially-Abundant Proteins (DAP)	Reference
Barley lines 004186 (S), 004223 (T)—shoot	20% PEG; 3 days water withholding	2DE MALDI-TOF, nanoLC-MS/MS	Up: V-ATPase, MDH, OEE (T), HSP70—up (T), down (S) Down: RubisCO activase (S)	[94]
Barley cv. Amulet—crown	30, 35% SWC (14 days)	2D-DIGE MALDI-TOF/TOF	105 DAP—82 identified—Up: GPX, GST, chitinase II Down: cdc48, GID1L2, OEE1,2	[83]
Alfalfa (*Medicago sativa*) cv. Magali—leaf	7 days water withholding	2DE ESI-LC-MS/MS	Up: 2Cys-Prx, 14-3-3; DHAR, SOD Down: mtATP synthase CF1β, PRK, SBP	[36]
Maize (*Zea mays*) lines F2, *Io*—leaf	0, 14 days water withholding	2DE Edman sequencing	Up: cystatin, COMT, ENO, MDH, PPase, β-1,3-glucosidase	[119]
Maize lines F2, *Io*—leaf	3,4,5 days water withholding	2DE Edman sequencing, nano LC ESI-MS/MS	Up: CCOMT, COMT, methionine synthase, SAMS	[133]
Maize line FR697—root elongation zone—cell wall (apoplast)	−0.03 MPa (control); −1.6 MPa (dehydration)	2DE HPLC-ESI-Q-TOF MS	Cell wall apoplast proteins: expnasin, XET, β-D-glucosidase	[135]
Maize genotypes CE704, 2023—leaf	6 days water withholding	2DE MALDI-TOF, iTRAQ	Several isoforms (HSP26, HSP70, 14-3-3)	[117]
Soybean (*Glycine max*) cv Taegwang—root	79, 62, 46, 29, 23% FWC (5 days); recovery: 85% FWC (4 days)	2DE MALDI-TOF	45 DAP (CCOMT, IFR; regulatory proteins (MADS-box TF KIP—down under stress, up under recovery)	[74]
Sugar beet (*Beta vulgaris*)—enotypes 7112, 7219-P6.9—leaf	157 days—field; irrigated (control), non-irrigated (drought)	2DE LC-MS/MS	157 DAP (20 identified—RubisCO, sHSP, NDPK, redox regulation Down: α-NAC)	[82]
Sunflower (*Helianthus annuus*) lines RGK21 (S), BGK329 (T)	Field experiment—water withholding at flowering stage	2DE nanoLC-MS/MS	Genotypic differences: APX, Cu/Zn-SOD; ENO, PGM—up in T, down in S; CHS—up in S, down in T	[33]

Table S1. *Cont.*

Plant Material	Treatment	Methods	Major Differentially-Abundant Proteins (DAP)	Reference
root				
Wild watermelon (*Citrullus lanatus*)—leaf	50%–60% FWC	2DE Edman sequencing	6 DAP—HSP70, vegetative storage protein	[115]
**Osmotic stress (PEG-6000)**				
Common wheat cv Yumai 34—leaf	Hoagland solution, 15% PEG-6000 (3 days); 0.5 mM SA pretreatment (3 days)	TCA/acetone; 2DE MALDI-TOF/TOF	82 (76 identified proteins), of which 35 SA-responsive proteins Up: 14-3-3; APX, GST, SA-responsive proteins: GS1c, GST1, PDI; ATP synthase CF1 α,β	[55]
Common wheat—spring wheats Abbondanza (T), Qingchun 38 (S)—leaf	PEG-6000 (−1 MPa; 72 h), recovery (24 h)	TCA/acetone; 2DE MALDI-TOF/TOF	38 (35 identified proteins) Up: GAPDH B; 26S proteasome, V-ATPase A Down: RubisCO LSU and SSU, GAPDH, TPI, AGPase (starch biosynthesis) Genotypic differences: Less PEG-affected proteins in T than S	[87]
Common wheat cv Hanxuan 10 (T) and Ningchun 47 (t)—seedling leaf	Hoagland solution, 20% PEG-6000 (−075 MPa) for 48 h	TCA/acetone/phenol; phosphopeptide enrichment via TiO_2 microcolumns; LC-MS/MS	173 (T) and 251 (t) phosphoproteins identified Phosphoproteins identified: signalling (SnRK2 kinase, protein phosphatase 2C, CDPK, calmodulin 2-2); transport (AQP, MSSP2; H^+-ATPase); LEA proteins (WCOR719, WCOR825, WRAB17)	[51]
Maize (*Zea mays*)—seedling leaf—membrane fraction	Hoagland solution, 16% PEG-6000 (8 h)	2DE MALDI-TOF	Up: 17 (RubisCO activase, ATP synthase CF1β, OEE1, ferredoxin-NADP reductase) Down: 18 (ATP synthase CF1α,γ; 50S ribosomal protein L28)	[84]
Soybean (*Glycine max*) cv Enrei—seedling root and hypocotyl plasma membrane fraction	10% PEG-6000 (2 days)	2DE, nanoLC-MS/MS	Up: calnexin, H^+-ATPase, ribosomal protein S10, TPI	[50]

Table S1. *Cont.*

Plant Material	Treatment	Methods	Major Differentially-Abundant Proteins (DAP)	Reference
Sunflower (*Helianthus annuus*) cv. Peredovick—leaf	PEG-6000: −0.05 MPa (control), −0.6 MPa (stress)	2DE MALDI-TOF	19 marker proteins—Up: CCOMT, FRK, Cu/Zn-SOD, GRP-RNA binding protein	[134]
**Waterlogging**				
Common wheat (*T. aestivum*) cv. Shiroganekomugi—seedling root	2 days waterlogging	2DE nanoLC-MS/MS	15 DAP—Up: methionine synthase, β-1,3-glucanase, β-glucosidase, chitinase III, ENO, MDH	[136]
Maize (*Zea mays*) A3237 (T), A3239 (S)—seedling root	7 days waterlogging	iTRAQ LC-MS/MS	3318 identified; 211 DAP—Up: ADH, NADP-ME, XET6, GST,	[100]
Soybean (*Glycine max*) cv. Enrei—root cell wall	2 days waterlogging		204 identified cell wall proteins—16 DAP Down: GLP, LOX, stem glycoprotein, Cu/Zn-SOD; pI change in copper amino oxidase	[123]
Tomato (*Lycopersicon esculentum*) cv Koma—leaf	2 cm water above soil surface (24, 48, 72 h)	2DE MALDI-TOF, ESI-MS/MS	52 DAP (33 identified—Up: MIPS; Down: ATP synthase CF1α,β)	[116]
**Salinity**				
Common wheat (*T. aestivum*) Jinan 177 (S), *T. aestivum* × *Thinopyrum ponticum* Shanrong 3 (T)—seedling root	½ Hoagland solution, 200 mM NaCl (24 h)	TCA/acetone; 2DE MALDI-TOF, MALDI-TOF/TOF	114 (110 identified—49 salt-responsive, 34 genotypic differences) Up: 14-3-3; Down: tubulin α-3 Genotypic differences: DEAD-box RNA helicase, DWARF3 (GA biosynthesis), eIF5A2, V-ATPase subunit E—higher in T; G-protein β subunit, ethylene receptor ETR1—higher in S	[54]

Table S1. *Cont.*

Plant Material	Treatment	Methods	Major Differentially-Abundant Proteins (DAP)	Reference
Common wheat cv. Calingiri, Janz (S), Wyalkatchem (T)—shoot mitochondrial fraction	200 mM NaCl (increase per 50 mM NaCl/day)	Isolation: PVP gradient; acetone extraction; 2D-DIGE LC-MS/MS	192 DAP (68 identified) Up: AOX, Mn-SOD, VDAC Down: CS, NDPK, outer mitochndrial membrane porin Genotypic differences: AOX, Mn-SOD—higher in T	[98]
Common wheat cv. Zhengmai 9023—leaf	1, 1.5, 2, 2.5% NaCl (2 days)	2D-DIGE Q-TOF-MS	125 DAP (52 identified—Up:26—ferritin, H^+-ATPase, GST, TPI Down: 21	[81]
Common wheat cvs. Jing-411 (T), Chinese Spring (S)—seedling root	0.5, 1.5, 2.5% NaCl (2 days)	2DE MALDI-TOF/TOF	198 DAP (144 identified genotypic differences: RubisCO LSU binding protein CPN60-α, PR10—higher in T than S	[58]
Common wheat cv. Keumgang—leaf chloroplast fraction	150 mM NaCl (1, 2, 3 days)	Isolation: Percoll gradient; TCA/acetone; 2DE LTQ-FTICR-MS	100 DAP (65 identified) Up: RubisCO, GAPDH, GDH, PDX1.2 and PDX1.3 Down: ATP synthase α,β,γ; V-ATPase	[85]
Common wheat (*T. aestivum*) cv Chinese Spring (S), *T. aestivum* × *Lophopyrum elongatum* amphiploid (T)—mitochondrial fraction (shoot, root)	200 mM NaCl (increase per 50 mM NaCl/day)	100% acetone (leaf), TCA/acetone (root); 2D-DIGE MALDI-TOF/TOF; HPLC Q-TOF MS/MS (peptide fingerprinting—genotypic differences)	55 root, 15 shoot differentially abundant proteins Organ-specific differences: aspartate aminotransferase, GDH (up in shoot, down in root) Genotypic differences: Mn-SOD, MDH, aconitase, SHMT, β-CAS - higher in T	[99]
Durum wheat (*T. durum*) cv. Ofanto—leaf	100 mM NaCl (2 days)	TCA/acetone 2DE MALDI-TOF	38 identified proteins Up (28): TPI; CPN60-β, RubisCO activase, carbonic anhydrase; osmolyte biosynthesis-related enzymes (glycine dehydrogenase, SAMS); COR; Cu/Zn-SOD Down (10): ALDO, PGK, RubisCO SSU, OEE1 precursor, β-glucosidase, ATP synthase CF1 α	[29]

Table S1. *Cont.*

Plant Material	Treatment	Methods	Major Differentially-Abundant Proteins (DAP)	Reference
Durum wheat cv. Waha—seed embryo and surrounding tissue	250 mM NaCl (42 h)—AsA priming (0.5 mM)	KCl (100 mM), acetone/ nano HPLC-MS	697 identified proteins—proteins involved in energy metabolism, protein metabolism, disease/defence, protein destination, storage—a positive effect of AsA priming on mitigation of salinity stress	[73,121]
Barley cv OUK305 (T), OUI743 (S)—root	200 mM NaCl (5 days)	40 mM Tris, 8 M urea, 4% CHAPS, 0.2% Bio-Lyte; 2DE nanoLC-ESI-MS/MS	6 differentially abundant proteins CCOMT, DHAR, GST (2 spots), POX, PR10—higher in T than S	[128]
Barley cv Morex (T), Steptoe (S)—root	100, 150 mM NaCl (13 days)	TCA/acetone; 2DE MALDI-TOF; nanoLC-ESI-Q-TOF MS/MS	39 differentially abundant proteins Up: LOX1, POX, SAMS, β-1,3-glucanase Down: IDI1, IDI2, IDS2, IDS3, Genotypic differences: class III POX, SAMS—higher in T; APX, MDAR—higher in S	[80]
Barley cv Afzal (T), L-527 (S)—leaf	300 mM NaCl (increase per 50 mM NaCl/day) 24 h	TCA/acetone; 2DE MALDI-TOF/TOF	117 DAP (22 identified proteins) Up:, PC, OEE2, PSI subunit VII (PsaC), PRK; 2-Cys Prx, Trx, GST, SOD; TPI, FBP ALDO—higher in T than S	[77]
Barley cv Afzal (T), L-527 (S)—leaf	300 mM NaCl (increase per 50 mM NaCl/day)—21 days	TCA/acetone; 2DE MALDI-TOF/TOF	44 DAP Up (43): RubisCO LSU, SSU, RubisCO activase, OEE2; NDPK; GLP; profilin; ribosomal protein L12, 30S ribosomal protein S1; translationally-controlled tumor protein homolog Genotypic differences: DHAR, Trx—higher in S	[76]
Barley cv Morex (T), Steptoe (S)—root	100, 150 mM NaCl (0, 1, 4, 7, 10 days)	TCA/acetone; 2DE MALDI-TOF; nanoLC-ESI-Q-TOF MS/MS	91 DAP (74 identified proteins) Genotypic differences: GLP3-7, GLP12, β-1,3-glucanase, ATP synthase CF1 β—higher in T; GLP5a, PR17—higher in S	[130]
Barley lines DH14 (S), DH187 (T)—root	100 mM NaCl (6 days)	2DE MALDI-TOF/TOF	Genotypic differences: annexin, TCTP, LOX, osmotin, V-ATPase—higher in T than S	[72]
Canola (*Brassica napus*) cvs Hyola 308 (T), Sarigol (S)—leaf	0, 175, 350 mM NaCl	2DE MALDI-TOF/TOF	44 DAP (T), 33 DAP (S) (Cu/Zn-SOD, PRK; PSBO2, ATP synthase CF1γ, 30S ribosomal protein S10; RubisCO LSU, SSU)	[67]

Table S1. *Cont.*

Plant Material	Treatment	Methods	Major Differentially-Abundant Proteins (DAP)	Reference
Grapevine (*Vitis vinifera*)—cv. Razegui—leaf, stem, root	100 mM NaCl (15 days)	2DE Edman sequencing	48 DAP—up: 32; down: 9; 7 newly induced—PR10	[127]
Maize (*Zea mays*)—hybrid SR12 (T)—leaf chloroplast fraction	1, 25 mM NaCl (1, 2, 4 h)	2DE MALDI-TOF	Up: 12 (ferredoxin-NADP reductase, 23 kDa PSII, FtsH-like; ATP synthase CF1ε); Down: 8 (ATP synthase CF1α, LHC *a*/*b* binding protein)	[48]
Maize hybrid SR12 (T)—root	25 mM NaCl (1 h)	2DE MALDI-TOF	Up:FRK, UDP-Glc transferase BX9, 2-Cys Prx; 14-3-3, XET Dephosphorylation: CaM, 40S ribosomal protein	[49]
**phosphoproteome**				
Pea (*Pisum sativum*) cv. Cutlass—root	75, 150 mM NaCl (28, 42, 56 days)	2DE ESI-Q-TOF-MS/MS	35 DAP—up: IFR, NDPK, PR10, SOD, Hsc82; H^+-ATPase, V-ATPase	[41]
Potato (*Solanum tuberosum*) cvs Concord (S), Kennebec-(T)—shoot	90 mM NaCl	2DE Edman sequencing	47 DAP: Up: calreticulin, TSI-1, osmotin-like, HSP Down: RubisCO, carbonic anhydrase, OEE; GS—more in S than T	[93]
Soybean (*Glycine max*) cv. Enrei—3-day-old seedling hypocotyl, root	100 mM NaCl (3 days)	2DE Edman sequencing; ESI-Q-TOF-MS/MS	7 DAP—up: β-conglycinin, bHLH protein, elicitor peptide; Down: protease inhibitor, lectin, stem 31 kDa glycoprotein precursor	[64]
Soybean cv. Enrei—leaf, hypocotyl, root	40 mM NaCl (7 days)	2DE MALDI-TOF	Leaf: 340 (19 identified), hypocotyl 330 (22 identified), root 235 (14 identified) Up: stem 31 kDa glycoprotein; Down: FRK, GAPDH	[95]
Tobacco (*Nicotiana tabacum*) cv. Petit Havana—leaf apoplast	100 mM NaCl (20 days)	2DE LC-MS/MS	20 DAP (LTP, peroxidase, chitinase, α-galactosidase, β-galactosidase, β-D-xylosidase)	[129]
Tobacco cv. Wisconsin—leaf	150 mM NaCl	2DE Edman sequencing, MALDI-TOF	18 DAP (Up: OEE2, RubisCO SSU; Down: stromal HSP70)	[92]

Table S1. *Cont.*

Plant Material	Treatment	Methods	Major Differentially-Abundant Proteins (DAP)	Reference
Tomato (*Lycopersicum esculentum*)—cvs Cervil, Super Marmande (S), Levovil, Roma (T)—root	100 mM NaCl (14 days)	2DE nanoLC-MS/MS	90 DAP; Up: HSP90; Down: V-ATPase Genotypic differences: Trx-H—increased in S and decreased in T TSI-1, PR10—in all genotypes, highest in Super Marmande	[124])
**Imbalances in Mineral Nutrients**				
**Boron**				
Barley Clipper (S) × Sahara (T) DH lines—leaf, root	1 mM H_3BO_3 (S), 5 mM H_3BO_3 (T) for 14 days	PBS pH 7.5, TCA/acetone; 2D-nanoLC-MS/MS iTRAQ	138 (leaf), 341 (root) identified proteins Up: IDS2, IDS3, methyl-thioribose kinase Leaf: PRK, PGK, PGM, ENO, PC, RubisCO activase, eEF1-α,β,γ; eEF-G, eEF-Tu; TLP; Cu/Zn-SOD; 50S ribosomal protein L3; 60S ribosomal protein L1 Root: CCOMT, class III POX, chitinase, 26S proteasome, β-1,3-glucanase; ATP synthase CF1 β, IDS2, IDS3; Hsp70; Hsc70; 40S ribosomal protein S5	[79]
White lupin (*Lupinus albus*) cv. Rio Major—leaf	Arnon solution with 0 µM B (deficient), 23.1 µM B (control)—28 days	2DE MALDI-TOF/TOF	23 DAP (9 identified)—PR1, β-1,3-glucanase, thamatin-like, expansin-like, class III chitinase	[39]
**Copper**				
Common wheat cv Yumai 34—eaf, root	100 µM $CuSO_4$ (3 days)	TCA/acetone; 2DE MS/MS	98 DAP (93 identified proteins—43 (leaf), 49 (root)) 36 Cu-responsive proteins Leaf: 14-3-3; MDH, TPI; PDI; V-ATPase A; ATP synthase CF1 α; carbonic anhydrase, RubisCO activase, PSI subunit VII (PsaC); Root: 14-3-3, translationally-controlled tumor protein; Hsp70, APX, GST, Cu/Zn-SOD, PR10; TPI, ATP synthase CF1 α; actin 1, tubulin	[37]

Table S1. *Cont.*

Plant Material	Treatment	Methods	Major Differentially-Abundant Proteins (DAP)	Reference
**Nitrogen**				
Common wheat cv Arche, Récital—leaf	2, 8, 20 mg N/plant/d for 60 days	TCA/acetone; 2DE LC-MS/MS	76 DAP (14 identified proteins) FBP ALDO, PGK, PGM, ENO2, MDH; RubisCO activase A, OEE1 (PsbO); 2-Cys Prx	[90]
**Heavy metal stress**				
**Cadmium**				
Indian mustard (*Brassica juncea*) line PI-173874—root	250 µM $CdCl_2$ (3 days)	2D-DIGE nanoLC-MS/MS; iTRAQ	2D-DIGE: 69 DAP (GST; APX;) iTRAQ: 130 DAP (GST; peroxidase; FE-SOD (up), Cu/Zn-SOD (down)—12% overlap between 2D-DIGE and iTRAQ	[31]
Flax (*Linum usitatissimum*)—cvs. Jitka, Tábor—hypocotyl cell suspension culture	10, 50, 100 µM $Cd(NO_3)_2$ for 48 h	2DE MALDI-TOF/TOF	14 DAP (ferritin, GS, lipocalin-1, α-tubulin 3, SAMS, HSP70, HSP83; IFR)	[34]
**Combined stress**				
**Osmotic stress or salinity** Common wheat (*T. aestivum*) Jinan 177 (S), *T. aestivum* × *Thinopyrum ponticum* Shanrong 3 (T)—root,	½ Hoagland solution 18% PEG-6000 or 200 mM NaCl (24 h)	TCA/acetone; 2DE MALDI-TOF/TOF	93 (root), 65 (leaf) differentially abundant proteins; 34 (root), 6 (leaf)—genotypic differences PEG: 38 root, 39 leaf; Salinity: 52 root, 52 leaf proteins PEG-specific proteins: ribosomal protein S8 (↓)Salt-specific proteins: importin α 1b (root), Genotypic diffrences: chl *a*/*b* binding apoprotein CP24 precursor, DWARF3—higher in T	[47]
**leaf**				
**Drought and heat** Barley—Syrian landrace Arta (T), Australian cv Keel (T)—leaf (heading stage)	Drought: 50% FWC (control), 15% FWC (stress) for 3 days Heat: 36 °C (4 h)	TCA/acetone; 2DE, 2D-DIGE MALDI-TOF/TOF	99 DAP Heat-up: FBP ALDO, chaperones, proteases, eEF-G, eIF4A, RubisCO activase B Genotypic differences (14 proteins): photosynthesis-related proteins (LHCII type III Lhcb3, OEE1 PsbO, RubisCO activase B)—higher in Keel than Arta	[91]

Table S1. *Cont.*

Plant Material	Treatment	Methods	Major Differentially-Abundant Proteins (DAP)	Reference
**Drought or waterlogging and cold** Winter common wheat cv. Yannong 19—leaf	Drought + LT: 35% FWC (7 days) Waterlogging + LT (7 days)	TCA/acetone; 2DE MALDI-TOF/TOF	32 identified proteins Up: DHAR, GR; Hsp70; Down: C metabolism-related proteins (glycolysis, TCA, Krebs cycle), RubisCO activase A, ATP synthase CF1 α,β	[143]
Heat or drought—Wheat cv. Vinjett—grain	Drought Heat: 32 °C (2 h)	2DE MALDI-TOF/TOF	Both drought and heat: α-amylase inhibitor, LEA, Prx, storage protein Heat only—up: HSP, 14-3-3	[144]
Cold; drought; herbicide (paraquat**)** Pea (*Pisum sativum*) cv. Green Feast—eaf mitochondrial fraction	Cold: 4 °C (36 h); Drought: 7 days water withholding; Paraquat: 662.5 mg/L	2DE, BN-PAGE, Q-TOF MS	DAP: GDC, SHMT, HSP70, HSP22, CF1 ATP synthase α,β,γ	[42]
**Drought, salinity** Grapevine (*Vitis vinifera*)—cvs Chardonnay, Cabernet Sauvignon—shoot	Drought:water withholding Salt: gradual increase 0 to 250 mM NaCl in 16 days	2DE MALDI-TOF/TOF	191 identified proteins—Up: PR10, Prx Genotypic differences: PPR	[66]
**Drought *vs.* flooding** Soybean (*Glycine max*) cv. Enrei—2-day-old seedling root	Drought: water withholding Flooding	nanoLC-MS/MS	Drought: 97 DAP (Up: cell wall lignification—SAMS) Flooding: 93 DAP (Up: 11 fermentation-related: ADH; Down: SAMS) Both stresses: glycolysis enzymes	[78]
